# Exploring changes to resident thriving and associated factors in Swedish nursing homes: A repeated cross‐sectional study

**DOI:** 10.1002/gps.5731

**Published:** 2022-05-18

**Authors:** Rebecca Baxter, Hugo Lövheim, Sabine Björk, Anders Sköldunger, David Edvardsson

**Affiliations:** ^1^ Department of Nursing Umeå University Umeå Sweden; ^2^ Center for Collaborative Palliative Care Department of Health and Caring Sciences Linnaeus University Växjö Sweden; ^3^ Department of Community Medicine and Rehabilitation Geriatric Medicine Umeå University Umeå Sweden; ^4^ Department of Public Health and Clinical Medicine Section of Sustainable Health Umeå University Umeå Sweden; ^5^ School of Nursing and Midwifery La Trobe University Melbourne Victoria Australia

**Keywords:** cognitive impairment, cross‐sectional study, long‐term care, neuropsychiatric symptoms, nursing home, Sweden, thriving

## Abstract

**Objective:**

This study aimed to explore changes to resident thriving in Swedish nursing homes over a 5‐year period and describe changes in associated factors.

**Methods:**

Cross‐sectional data were collected from a randomised sample of Swedish nursing homes in 2013/2014 (baseline) and 2018/2019 (follow‐up). Descriptive statistics, independent samples *t*‐tests, and chi squared tests were used to statistically evaluate differences between the samples. Simple and multiple linear regression analyses were used to explore associations between thriving and the study variables.

**Results:**

Resident characteristics were relatively consistent between the full baseline (*N* = 4831) and follow‐up (*N* = 3894) samples. Within a sub‐sample of nursing homes that participated in both data collections mean thriving scores were found to have increased from 152.9 to 155.2 (*p ≤ *0.003; *d *=0.09) and overall neuropsychiatric index scores had decreased from 16.0 to 14.3 (*p ≤ *0.004; *d *=0.09), as had the prevalence of several neuropsychiatric symptoms. Thriving was found to have a positive association with the neuropsychiatric symptom of elation/euphoria, and negative associations with the symptoms of aggression/agitation, depression/dysphoria, apathy, and irritability.

**Conclusions:**

The results show an increase in overall thriving scores and a decrease in overall neuropsychiatric scores between baseline and follow‐up. This study confirmed associations between thriving and certain neuropsychiatric symptoms and established comparative knowledge regarding changes in resident thriving, characteristics, and symptom prevalence. These findings could inform future care and organisational policies to support thriving in nursing homes, particularly among residents at risk of lower thriving due to cognitive impairment or neuropsychiatric symptoms.

## Key points

- Thriving scores increased significantly between baseline and follow‐up in this sample of Swedish nursing home residents.
- Residents' overall neuropsychiatric‐index scores decreased significantly, as did the prevalence of most neuropsychiatric symptoms.
- Associations between resident thriving, cognitive impairment, and neuropsychiatric symptoms were confirmed and elucidated.
- Further research is needed to explore the impact of person‐centred interventions to improve resident outcomes over time, particularly among residents at risk of lower thriving.

## 1 INTRODUCTION

Preservation of good standards of living, quality of life, and societal participation is mandated by law for older people in Sweden (i.e., the Social Services Act, 2001:453); yet, for people living in nursing homes monitoring of outcomes continues to focus on measuring negative symptoms related to decline in older age (i.e., falls, pressure areas, medication consumption), with little in the way of exploring positive salutogenic outcomes. In recent years, the concept of thriving has come to the fore as a useful way to measure and understand experiences of place‐related well‐being among older persons living in nursing homes because it is not necessarily contingent upon a person's physical, cognitive, or functional status.^1^, ^2^

Experiences of thriving are theorised to emerge from balanced interactions between a person and their human and non‐human environment.^3^ In Scandinavia, the term thriving is used to describe lived experiences of enjoying and/or being in a specific place or space.^4^ Among older adults living in nursing homes, thriving is said to comprise of two core aspects: the residents' attitude towards living in a long‐term care facility and the quality of care and caregivers; and five peripheral aspects related to the person's relationships, activities, and lived‐environment.^1^, ^2^ While quality of life and general well‐being encompass elements such as health and physical conditioning, thriving is said to focus more explicitly on the person's interaction with, and adjustment to, their social, relational, and institutional environment—irrespective of (ill) health or function.^1^, ^2^, ^5^

Nursing home demographics are changing with the ageing population, and previous research has found that residents who thrive generally have higher levels of cognitive and activity functioning, lower prevalence of neuropsychiatric symptoms, and higher overall quality of life.^6^, ^7^, ^8^ It seems important to explicate associations between these characteristics and outcomes over time, but estimations regarding the prevalence of cognitive impairment, neuropsychiatric symptoms, and changes in activities of daily living in nursing homes are highly variable between studies and contexts. Information regarding trends for cognitive impairment in Sweden is wide‐ranging, with some studies reporting stable or declining incidence of dementia,^9^, ^10^, ^11^, ^12^ and others indicating increasing prevalence of dementia.^13^ The Swedish Register for Behavioural and Psychological Symptoms of Dementia^14^ (BPSD) estimates that 70% of nursing home residents are cognitively impaired. This figure is supported by a recent study that found 42% of nursing home residents had a formal diagnosis of cognitive impairment and 72% had some level of (undiagnosed) impairment, indicating that such symptoms are not easily or readily recognised.^15^ The prevalence of contiguous neuropsychiatric symptoms is estimated to be markedly higher, with Selbæk and colleagues^16^ finding 82% of nursing home residents with dementia had at least one neuropsychiatric symptom, the most frequent being agitation and apathy. Other neuropsychiatric symptoms are said to be common among residents, with high rates of depression (i.e., National Board of Health and Welfare^17^), agitation (i.e., Schmüdderich et al.^18^) and anxiety (i.e., Creighton et al.^19^). Diminished ability to perform personal activities of daily living has also been associated with more severe dementia, presence of neuropsychiatric symptoms, and lower quality of life.^20^, ^21^ In this way, it seems necessary to ascertain not only the extent to which nursing home resident demographics, symptoms, and characteristics have changed over time, but how these changes have impacted residents' experiences of well‐being in their lived environment.^22^, ^23^, ^24^

In recent years, changes have been made to policy and practice with regards to symptom management, medication administration, and person‐centred care interventions^14^, ^25^, ^26^; but the potential downstream effects of these interventions have had limited investigation and evaluation from a salutogenic and health‐promoting perspective. Examining changes that may have occurred over time with regards to thriving in Swedish nursing homes could therefore inform and support health‐promoting outcomes across all levels of care, policy, and practice. Thus, the aim of this study was to explore changes to thriving in Swedish nursing homes over a 5‐year period and describe changes in associated factors.

## 2 METHODS

### 2.1 Data and design

A repeated cross‐sectional design was used to collect data from Swedish nursing homes over a 5‐year period. While the participants (both staff and residents) may have changed during this time, inferences of change at population‐level are possible as the same overall groups were sampled, including a sub‐sample of nursing homes that participated in both data collections. This study is part of the Swedish National Inventory of Care and Health in Residential Aged Care (SWENIS) study, formed to initiate long‐term monitoring of health and care in a nationally representative sample of older people living Swedish nursing homes.^27^ The SWENIS I baseline data collection took place from November 2013 to September 2014, and the SWENIS II follow‐up data collection took place from November 2018 to May 2019. Data were collected using hard‐copy paper surveys that were completed by nursing home staff as proxy‐raters for residents. As this study was interested in population‐level monitoring, participants were not identified to individual‐level. The survey included items regarding demographic information, care characteristics, cognitive impairment, neuropsychiatric symptoms, activities of daily living, and thriving. The Swedish Regional Ethical Review Board approved the overall SWENIS I (2013/269‐31) and SWENIS II studies (2018/145‐31).

Of Sweden's 290 municipalities, 60 were randomly selected for invitation to the SWENIS I study. The municipal manager was contacted for permission to undertake research in their municipality. Forty‐seven municipal managers agreed initially, but five municipalities did not respond to the request to provide the names and contact details for the nursing homes in their municipalities and five municipalities withdrew. Next, 202 nursing homes in 37 municipalities were contacted and given verbal and written information about the study. A total of 4831 completed surveys (response rate, 70%) were received from 172 nursing homes in 35 municipalities. A similar procedure was followed for the SWENIS II follow‐up data collection. The 35 municipalities that took part in SWENIS I were offered the opportunity to participate in the SWENIS II study, and an additional 25 randomised municipalities were also invited to bring the total number of municipalities back to 60. Forty‐nine municipal managers agreed initially, and 315 nursing homes were contacted for participation; however, four municipalities withdrew and two dropped out during this process. The final SWENIS II sample comprised of 3894 proxy‐rated resident surveys (response rate, 55%) from 187 nursing homes in 43 municipalities (28 municipalities from SWENIS I, 15 new municipalities).

### 2.2 Measures

#### 2.2.1 The Thriving of Older People Assessment Scale (TOPAS)

The TOPAS was used to measure thriving. The TOPAS comprises 32 item statements that are scored 1 (No, I disagree completely) to 6 (Yes, I agree completely) for a possible sum score of 32–192 where a higher score indicates higher thriving.^28^, ^29^ The TOPAS contains five domains: (1) the resident's attitude (towards living in the nursing home) (4 items), (2) the quality of care and caregivers (11 items), (3) resident engagement and peer relationships (8 items), (4) keeping in touch with people and places (4 items), and (5) qualities in the physical environment (5 items).^28^, ^29^ Previous studies have reported satisfactory reliability and validity for the TOPAS and endorsed the scale for self‐ and proxy‐rated use in nursing homes.^28^, ^29^, ^30^

#### 2.2.2 The Gottfries Cognitive Scale (GCS)

The GCS was used to measure cognitive function.^31^, ^32^ The GCS consists of 27 dichotomous statements regarding personal, environmental, and abstract orientation. Each statement is answered ‘yes’ (1) or ‘no’ (0), where scores below 24 indicate cognitive impairment. The sum scores can be further divided into subgroups of mild impairment (score, 23–16), moderate impairment (score, 15–8), and severe impairment (score, 7–0).^33^ The scale has been validated for proxy‐rated use in nursing homes.^32^

#### 2.2.3 The Katz Index of Independence in Activities of Daily Living (Katz‐ADL)

The Katz‐ADL was used to assess activity function.^34^ Six activity statements are assessed based on the level of dependence or independence required to complete personal ADL tasks: bathing, dressing, transferring, toileting, eating, and continence. Scores range from 0 (dependent in ADL) to 6 (independent in ADL), where a higher score indicates greater independence in ADL.^34^ Dependence in more than three activities was interpreted to be indicative of functional dependence.^35^

#### 2.2.4 The Neuropsychiatric Inventory – Nursing Home (NPI‐NH)

The NPI‐NH was used to measure psychological and behavioural neuropsychiatric symptoms.^36^, ^37^ The NPI‐NH contains 12 items to explore the frequency and severity of neuropsychiatric symptom domains, namely: delusions, hallucinations, agitation, depression, anxiety, euphoria, apathy, inhibition, irritability, abnormal motor behaviour, nocturnal anxiety, and appetite. Frequency is scored 0 (never) to 4 (very often), and severity is scored 1 (mild) to 3 (severe), where higher total scores indicate higher frequency and severity of neuropsychiatric symptoms.^37^

### 2.3 Statistical analysis

Descriptive statistics were used to calculate sample characteristics and measures of central tendency for the full SWENIS I and II samples. To maintain comparability between the samples, a sub‐sample of nursing homes that participated in both data collections was selected for further analysis. *T*‐tests and chi‐squared tests were undertaken to determine the significance of overall differences between the SWENIS I and SWENIS II sub‐samples. To confirm the statistical assumptions to support linear regression were met, normality and linearity were assessed by examining the histograms, scatterplots, and values for skewness (±2) and kurtosis (±1). To explore variables associated with thriving, a multiple linear regression was calculated with the dependent variable of thriving (TOPAS score) and the independent variables of neuropsychiatric symptoms (controlling for sex, age, activity function, and cognitive function). Given that lower cognitive functioning has been associated with lower thriving (i.e., Björk et al.^7^, Patomella et al.^8^), the output was separated into cognitive groups according to GCS score (i.e., none, mild, moderate, and severe). Statistical significance for all analyses were defined at *p* < 0.05 and effect sizes (Cohen's *d*) were calculated for total scale scores that indicated statistical significance.^38^ No imputations were used in the full SWENIS I and SWENIS II samples. In the sub‐samples, missing data for up to three items in the TOPAS and missing data for up to one item in the NPI‐NH were replaced with the mean value of the individual for the total scale,^39^ and missing data for up to three items in the GCS were imputed with 0.5.^32^ Data were analysed using IBM SPSS version 25 for Windows (IBM Corporation).

## 3 RESULTS

The proxy‐raters in SWENIS I were predominantly female (94%), enrolled nurses (84%), and were generally well acquainted with the resident in question with 57% reporting daily interactions and 42% reporting weekly interactions. Likewise, in SWENIS II the proxy‐raters were mostly female (92%), enrolled nurses (86%), and were well acquainted with the resident they were rating with 61% of proxy‐raters reporting daily interactions and 38% reporting weekly interactions.

In the full SWENIS I sample (*N* = 4831), most residents were female (68%) with a mean age of 85.5 years (±7.8 years) and an average length of stay of 30 months (±32 months) (Table 1). Around two‐thirds (67%) of residents were rated as having some form of cognitive impairment and 56% were rated as dependent in activities of daily living. In the full SWENIS II sample (*N* = 3894), residents were also mostly female (65%) with a mean age of 85.3 years (±8.4 years) and an average length of stay of 32 months (±35 months). The majority of residents (66%) were rated as having cognitive impairment and 56% were rated as dependent in activities of daily living. The mean scores for the GCS, NPI‐NH, Katz‐ADL, and TOPAS scales in the full SWENIS I and II samples are reported in Table 1.

**TABLE 1:** Characteristics of the full SWENIS I and SWENIS II samples and mean values of the study variables

	SWENIS I Full sample		SWENIS II Full sample	
	Mean (SD)	*N* (%)	Mean (SD)	*N* (%)
Total		4831		3894
Age	85.5 (7.8)		85.3 (8.4)	
Sex				
Female		3239 (67.8)		2515 (65.3)
Male		1538 (32.2)		1334 (34.3)
Length of stay (months)	30.4 (32.0)		32.2 (34.6)	
GCS score	17.3 (8.3)		17.5 (8.3)	
Cognitive impairment (any)		2827 (66.6)		2267 (66.0)
None		1418 (33.4)		1169 (34.0)
Mild		1067 (25.1)		869 (25.3)
Moderate		1092 (25.7)		875 (25.5)
Severe		668 (15.7)		523 (15.2)
Katz ADL score	2.9 (2.1)		3.0 (2.2)	
Dependent in ADL		2526 (56.3)		2022 (55.9)
NPI‐NH score	17.3 (20.5)		14.9 (18.6)	
TOPAS score	152.2 (25.0)		154.5 (25.2)	

Abbreviations: GCS, Gottfries cognitive Scale; Katz‐ADL, Katz Index of Independence in Activities of Daily Living; NPI‐NH, Neuropsychiatric Inventory – Nursing Home; SWENIS, Swedish National Inventory of Care and Health in Residential Aged Care; TOPAS, Thriving of Older People Assessment Scale.

Within a sub‐sample of 91 nursing homes that participated in both SWENIS I (*n* = 2559) and SWENIS II (*n* = 2040), no significant differences were found in resident age, sex, activity function, or cognitive status (Table 2). The mean TOPAS score was found to have increased from 152.9 to 155.2 (*p* = <0.003; *d* = 0.09), while the mean score for the NPI‐NH decreased from 16.0 to 14.3 (*p* = <0.004; *d* = 0.09). In the sub‐sample, 10.7% of participants in SWENIS I scored 120 or less on the TOPAS, while in SWENIS II only 8.6% of participants scored 120 or less (*p* = <0.019) (not shown). A simple linear regression model with thriving (i.e., TOPAS score) as the dependent variable (adjusting for sex, age, activity function and cognitive function) confirmed that individuals in the SWENIS II sub‐sample scored an average of 2.7 points higher on the TOPAS than those in SWENIS I (*p* ≤ 0.001) (not shown).

**TABLE 2:** Characteristics of the nursing home sub‐samples from SWENIS I and SWENIS II and mean values of the study variables

	SWENIS I		SWENIS II		
	Sub‐sample		Sub‐sample		*p*
	Mean (SD)	*N* (%)	Mean (SD)	*N* (%)	
Total		2559		2040	
Age	85.7 (7.7)		85.5 (8.1)		0.562
Sex					
Female		1731 (68.3)		1380 (68.1)	
Male		804 (31.7)		645 (31.9)	0.922
Length of stay (months)	30.1 (31.1)		33.0 (33.6)		0.010
GCS score	17.2 (8.2)		16.9 (8.2)		0.243
Cognitive impairment (any)		1717 (68.5)		1410 (70.5)	0.140
None		796 (32.2)		593 (30.0)	0.135
Mild		649 (26.2)		526 (26.6)	0.744
Moderate		648 (26.2)		514 (26.0)	0.922
Severe		380 (15.4)		344 (17.4)	0.063
Katz ADL score	2.9 (2.1)		2.9 (2.1)		0.436
Dependent in ADL		1342 (56.6)		1080 (56.9)	0.816
NPI‐NH	16.0 (19.9)		14.3 (17.2)		0.004
TOPAS	152.9 (25.2)		155.2 (24.8)		0.003

Abbreviations: GCS, Gottfries Cognitive Scale; Katz‐ADL, Katz Index of Independence in Activities of Daily Living; NPI‐NH, Neuropsychiatric Inventory – Nursing Home; SWENIS, Swedish National Inventory of Care and Health in Residential Aged Care; TOPAS, Thriving of Older People Assessment Scale.

Examination of neuropsychiatric symptom prevalence in the SWENIS I and SWENIS II sub‐samples showed that only aggression/agitation increased significantly (*p* ≤ 0.020) between the two time points (Table 3). While the prevalence of anxiety (*p* ≤ 0.005), apathy (*p* ≤ 0.007), sleep/night behaviours (*p* ≤ 0.011), and appetite/eating changes (*p* ≤ 0.023) decreased significantly. The symptom with the highest overall prevalence was depression/dysphoria (SWENIS I, 56.5%; SWENIS II, 54.6%) and the lowest was euphoria (SWENIS I, 21.2%; SWENIS II, 21.6%).

**TABLE 3:** Comparison of neuropsychiatric symptom prevalence between the SWENIS I and SWENIS II sub‐samples of matching nursing homes

	SWENIS I Sub sample	SWENIS II Sub‐sample	*p*
	% (*N*)	% (*N*)	
Delusions	34.8 (850)	33.9 (671)	0.524
Hallucinations	29.1 (710)	28.1 (557)	0.484
Aggression/agitation	41.8 (1022)	45.3 (898)	**0.020**
Depression/dysphoria	56.5 (1386)	54.6 (1084)	0.219
Anxiety	45.2 (1111)	41.1 (815)	**0**.**005**
Euphoria	21.2 (521)	21.6 (429)	0.729
Apathy	48.7 (1196)	44.7 (882)	**0**.**007**
Disinhibition	28.6 (702)	27.3 (541)	0.349
Irritability	48.7 (1196)	49.4 (976)	0.631
Aberrant motor behaviour	32.3 (792)	32.2 (637)	0.987
Sleep/night behaviours	40.7 (998)	37.0 (734)	**0**.**011**
Appetite/eating changes	41.6 (1003)	38.2 (742)	**0**.**023**

*Note*: *p* values derived from *t*‐tests; Bold numbers indicate significant associations (*p* < 0.05).

Abbreviation: SWENIS, Swedish National Inventory of Care and Health in Residential Aged Care.

A multiple linear regression model was calculated to explore associations between thriving (TOPAS score; dependent variable) and neuropsychiatric symptoms (adjusting for sex, age, activity function, and cognitive function). The output was separated into cognitive groups according to GCS score (i.e., none, mild, moderate, and severe). As shown in Table 4, five neuropsychiatric symptom variables showed the strongest consistent associations to thriving across the cognitive groups and samples. Thriving showed a positive association with symptoms of elation/euphoria, and a negative association with symptoms of aggression/agitation, depression/dysphoria, apathy, and irritability. Based on the adjusted *R* ^2^, among those with more severe cognitive impairment there was a weaker association with thriving and neuropsychiatric symptoms, and a stronger association among those with no, mild, or moderate cognitive impairment.

**TABLE 4:** Factors associated with thriving (TOPAS score; dependent variable) in relation to neuropsychiatric symptoms within the SWENIS I and SWENIS II sub‐samples—presented by level of cognitive impairment (CI) (i.e., none, mild, moderate, or severe)

	SWENIS I Sub‐sample				SWENIS II Sub‐sample			
	No CI *β* (*p*)	Mild CI *β* (*p*)	Mod CI *β* (*p*)	Sev CI *β* (*p*)	No CI *β* (*p*)	Mild CI *β* (*p*)	Mod CI *β* (*p*)	Sev CI *β* (*p*)
Delusions	−0.084 (0.202)	−0.118 (0.055)	−0.028 (0.626)	−0.075 (0.471)	−0.118 (0.063)	0.088 (0.151)	−0.065 (0.362)	**−0.217** (**0**.**021**)
Hallucinations	0.053 (0.459)	−0.078 (0.176)	0.104 (0.054)	0.023 (0.817)	**0.138** (**0**.**010**)	−0.050 (0.403)	0.003 (0.964)	0.096 (0.336)
Aggression/Agitation	**−0.212** (**0.001**)	−0.047 (0.451)	**−0.199** (**0**.**003**)	0.032 (0.770)	**−0.122** (**0**.**047**)	−0.112 (0.074)	**−0.158 (0.027)**	−0.193 (0.063)
Depression/Dysphoria	**−0.269** (**0**.**001**)	**−0.218** (**0**.**001**)	**−0.261** (**0**.**001**)	−0.061 (0.444)	**−0.164** (**0**.**013**)	**−0.123** (**0**.**040**)	**−0.210 (0.002)**	−0.106 (0.231)
Anxiety	0.044 (0.466)	0.027 (0.615)	−0.085 (0.146)	−0.035 (0.650)	0.089 (0.132)	−0.100 (0.106)	0.043 (0.525)	−0.028 (0.759)
Elation/Euphoria	**0.149** (**0.006**)	**0.166** (**0.001**)	**0.155** (**0.001**)	**0.150** (**0**.**038**)	−0.063 (0.243)	**0.138** (**0**.**011**)	−0.007 (0.906)	**0.192** (**0**.**011**)
Apathy	−0.094 (0.062)	**−0.156** (**0**.**001**)	**−0.142** (**0**.**004**)	**−0.191** (**0**.**009**)	**−0.173** (**0**.**001**)	**−0.178** (**0**.**001**)	−0.069 (0.228)	**−0.233** (**0**.**003**)
Disinhibition	−0.083 (0.231)	−0.093 (0.129)	−0.034 (0.563)	−0.154 (0.100)	0.003 (0.954)	−0.051 (0.430)	−0.015 (0.825)	0.056 (0.557)
Irritability	0.044 (0.523)	**−0.147** (**0**.**040**)	−0.075 (0.300)	−0.051 (0.681)	**−0.316** (**0**.**001**)	**−0.167** (**0**.**018**)	**−0.170 (0.025)**	0.063 (0.644)
Aberrant motor behaviour	0.009 (0.874)	−0.068 (0.192)	**0.108** (**0**.**049**)	−0.037 (0.630)	0.049 (0.302)	−0.004 (0.944)	−0.049 (0.439)	0.087 (0.293)
Sleep/night behaviours	0.044 (0.459)	0.038 (0.445)	−0.045 (0.410)	0.050 (0.485)	−0.069 (0.179)	0.024 (0.659)	−0.038 (0.513)	−0.077 (0.322)
Appetite/eating changes	−0.030 (0.527)	−0.006 (0.902)	−0.054 (0.259)	**−0.187** (**0**.**010**)	0.026 (0.602)	**−0.107** (**0**.**034**)	0.031 (0.579)	−0.007 (0.922)
Adjusted *R* ^2^	0.193	0.303	0.264	0.158	0.377	0.240	0.231	0.138

*Note*: Bold numbers indicate significant associations (*p* < 0.05).

Abbreviation: SWENIS, Swedish National Inventory of Care and Health in Residential Aged Care.

## 4 DISCUSSION

This study aimed to explore changes to thriving in Swedish nursing homes over a 5‐year period and describe changes in associated factors. Nursing home resident characteristics were reasonably consistent in the SWENIS I and SWENIS II full and sub‐samples, with no significant differences found in age, sex, cognitive function, or activity function. Mean thriving scores were found to have significantly increased, while neuropsychiatric‐index scores and the overall prevalence of most neuropsychiatric symptoms within the SWENIS I and II sub‐samples significantly decreased; however, the clinical and experiential impact of such changes require further consideration.

In Björk et al.'s^7^ study (derived from the baseline SWENIS I dataset), residents who were rated as having cognitive impairment were found to have lower thriving and higher prevalence of neuropsychiatric symptoms than cognitively intact residents. This is consistent with the larger body of research linking higher perceived frequency of neuropsychiatric symptoms, lower activity function, and lower cognitive functioning with lower levels of thriving, well‐being, and/or quality of life among nursing home residents. The present study confirms these relationships, and the negative associations between thriving and agitation, depressive symptoms, apathy, and irritability were still largely present in SWENIS II. Likewise, euphoria continued to demonstrate a positive association with thriving across both time‐points. Severe cognitive impairment had a weaker association to thriving across most neuropsychiatric symptoms, while the association with thriving was stronger among those with no, mild, or moderate cognitive impairment.

The overall prevalence of neuropsychiatric symptoms was found to have decreased, with only the symptom of aggression/agitation reporting a statistically significant increase. The least prevalent symptoms were hallucinations, euphoria, and disinhibition, and the symptoms with the highest prevalence were depression/dysphoria, aggression/agitation, and irritability. This seems to be in line with overall trends in BPSD for people living in Swedish nursing homes, with studies indicating declining or stable prevalence of most symptoms possibly due to changes in medication and treatment strategies.^44^, ^45^, ^46^ These findings are important to consider as past research has linked negative experiences of neuropsychiatric symptoms with greater unmet needs, feelings of distress and discomfort, and lower self‐ and proxy‐ rated quality of life.^41^, ^42^, ^43^, ^47^, ^48^ Moreover, the presence of moderate to severe neuropsychiatric symptoms has been associated with higher resident mortality, underscoring the importance of recurrent assessments and early intervention for persons exhibiting such symptoms.^49^

A major change that occurred between baseline and follow‐up was the implementation of a BPSD diagnostic checklist in 2018 (National Board of Health and Welfare^50^, ^51^; Swedish Register for BPSD^14^). The checklist requires clinicians to identify person‐centred alternatives as a first‐line response to alleviate neuropsychiatric symptoms, such as ensuring the resident has had sufficient food, sleep, activities, or socialisation, as well as identification of possible contributors or triggers, such as pain or acute illness.^14^ Managing these symptoms using structured working methods has been shown to reduce BPSD and improve quality of life for persons with dementia.^14^ Data from the Register for BPSD^14^ indicates that all Swedish municipalities are connected to the register, and that uptake of education and reporting increased between 2014 and 2019. Furthermore, since 2015 the Swedish Government has worked to prioritise and disseminate knowledge about person‐centred care to all of Sweden's municipalities for integration into healthcare services.^25^, ^26^ Person‐centred intervention strategies have been endorsed as having the potential to mitigate possible triggers for neuropsychiatric symptoms by optimising the social and physical environment to meet the needs of the individual person.^52^, ^53^, ^54^ These initiatives seem highly relevant to the concept of thriving as they link personal, relational, and environmental aspects to care experiences and outcomes; perhaps indicating that thriving could be useful for persons experiencing neuropsychiatric symptoms as it acknowledges the inherent connection between the lived environment and perceived well‐being.^5^

One tentative interpretation of these findings is that the changes that have been implemented over the last 5 years (i.e., medical, pharmacological, and person‐centred care interventions) may have contributed to a decrease in prevalence of neuropsychiatric symptoms and could be cautiously understood to have had a positive impact on resident thriving. Although, given the marginal effect sizes repeated follow‐up studies are required to confirm and elucidate the clinical and/or experiential impact of these changes at both population and individual levels. Thus, while statistical improvements to thriving and neuropsychiatric symptoms are encouraging, if residents, relatives, staff, and organisations do not likewise *experience* these changes to be positive then further innovation seems necessary to meaningfully enhance thriving in nursing homes.

### 4.1 Limitations

Due to the repeated cross‐sectional design of this study causality cannot be inferred between variables and the results may have been influenced by cohort effects. Residents and staff are likely to have changed in the last 5 years; thus, repeated measurement of resident characteristics, symptoms, and outcomes at individual‐level could be used to identify resident groups that require targeted interventions, environmental support, or organisational resources. The difference in response rates between the full SWENIS I and SWENIS II samples may have been impacted by changes to European Union General Data Protection Regulations (GDPR) that were highly publicised at the time of follow‐up data collection, but as reasons for non‐participation were not explored it is impossible to draw conclusions surrounding potential differences in response rates, non‐response bias, or non‐response error. When interpreting the results, the risk of type 1 error or random significances must be considered. The results of any single significant difference should be interpreted with caution and be confirmed in other studies. The use of nursing home staff as resident proxy‐raters may have influenced these findings as poor scores could be perceived to reflect negatively on their professional work. Nevertheless, previous studies have established that the TOPAS is valid and reliable to measure self‐ and proxy‐ rated thriving (i.e., Bergland et al.^28^, ^29^). Due to high levels of cognitive impairment within the samples the use of proxy‐raters could be viewed as a strength since the surveys were completed by staff who knew the resident well and had insight into their everyday care, activities, and relationships. Finally, it is possible that other variables that influence thriving were not included in this study, these should be explored in future research.

## 5 CONCLUSIONS AND IMPLICATIONS

This study found that thriving scores had significantly increased over a 5‐year period in a nationally representative sample of Swedish nursing home residents. Neuropsychiatric‐index scores and the prevalence of most neuropsychiatric symptoms were found to have decreased significantly. Associations were confirmed and elucidated between resident thriving, level of cognitive impairment, and neuropsychiatric symptoms. It seems essential to monitor resident outcomes over time in order to evaluate and improve the planning, organisation, and quality of nursing home care. This study adds to the thriving literature by establishing baseline and follow‐up changes to thriving in nursing homes and further illuminating associations between thriving and specific resident variables. Future research is required to explicate other variables that may influence thriving, as well as explore possible effects of person‐centred care interventions to support and promote thriving at individual‐level, particularly among persons and populations groups at risk of lower thriving.

## CONFLICT OF INTEREST

The authors declared that they have no conflicts of interest to this work.

## ETHICS STATEMENT

The Swedish Regional Ethical Review Board approved the overall SWENIS I (2013/269‐31) and SWENIS II (2018/145‐31) studies.

## Data Availability

The data that support the findings of this study may be available from the corresponding author, upon reasonable request.
